# Neonatal Seizure Detection Based on Spatiotemporal Feature Decoupling and Domain-Adversarial Learning

**DOI:** 10.3390/s26030938

**Published:** 2026-02-01

**Authors:** Tiannuo Xu, Wei Zheng

**Affiliations:** Ocean College, Jiangsu University of Science and Technology, Zhenjiang 212100, China

**Keywords:** neonatal seizure detection, EEG signals, domain-adversarial learning, feature decoupling, cross-subject generalization

## Abstract

Neonatal seizures are a critical early indicator of neurological injury, yet effective automated detection is challenged by significant inter-subject variability in electroencephalogram (EEG) signals. To address this generalization gap, this study introduces the Domain-Adversarial Spatiotemporal Network (DA-STNet) for robust cross-subject seizure detection. Utilizing Short-Time Fourier Transform (STFT) spectrograms, the architecture employs a hierarchical backbone comprising a Channel-Independent CNN (CI-CNN) for local texture extraction, a Spatial Bidirectional Long Short-Term Memory (Bi-LSTM) for modeling topological dependencies, and Attention Pooling to dynamically prioritize pathological channels while suppressing noise. Crucially, a Gradient Reversal Layer (GRL) is integrated to enforce domain-adversarial training, decoupling pathological features from subject-specific identity to ensure domain invariance. Under rigorous 5-fold cross-validation, the model achieves State-of-the-Art performance with an average Area Under the Curve (AUC) of 0.9998 and an F1-score of 0.9952. Data scaling experiments further reveal that optimal generalization is attainable using only 80% of source data, highlighting the model’s superior data efficiency. These findings demonstrate the proposed method’s capability to reduce reliance on extensive clinical annotations while maintaining high diagnostic precision in complex clinical scenarios.

## 1. Introduction

Neonatal seizures represent the most frequent neurological emergency during the neonatal period and often serve as an early indicator of severe injury to the central nervous system (CNS). As noted by Kaminiów et al. [1], hypoxic–ischemic encephalopathy (HIE), ischemic stroke, intracranial hemorrhage, and CNS infections are the most prevalent etiologies causing acute brain injury and subsequent seizures. Failure to timely detect and intervene in such abnormal neuronal electrical activity can precipitate irreversible brain damage, permanent neurodevelopmental impairments, or even mortality. Distinct from adult epilepsy, the clinical manifestations of neonatal seizures are frequently subtle and occult. They often present merely as minute limb movements, apnea, or exist in a state of “electrographic-only seizures” (clinical silence), resulting in a high rate of missed diagnosis when relying solely on clinical observation. Consequently, within the Neonatal Intensive Care Unit (NICU), prompt and accurate detection followed by targeted therapeutic intervention is paramount for mitigating long-term adverse sequelae. Although continuous EEG (cEEG) monitoring is universally recognized as the “gold standard” for diagnosis, its interpretation is heavily reliant on professionally trained neurophysiologists. The scarcity of such expert resources creates a bottleneck for continuous 24/7 monitoring, highlighting an urgent need for high-precision, automated computer-aided diagnosis systems to bridge this gap.

In recent years, artificial intelligence has achieved breakthrough advancements in biomedical signal processing, comprehensively shifting the research paradigm from traditional manual feature engineering to end-to-end deep representation learning. Convolutional Neural Networks (CNNs) [2] have been widely adopted due to their superior feature extraction capabilities. For instance, the LMA-EEGNet proposed by Zhou et al. [3] introduces dilated depth-wise separable convolutions, which significantly reduce computational complexity while maintaining high detection accuracy, thereby enabling real-time monitoring on edge devices. To address the limitations of CNNs in capturing cerebral spatial topological structures, Graph Neural Networks (GNNs) have emerged as a prominent research hotspot [4]. The ST-GAT model developed by Raeisi et al. [5] utilizes graph attention mechanisms to dynamically model the connection strength between electrodes, effectively revealing abnormal brain network flows during seizures. Concurrently, to tackle the issue of long-term temporal dependencies in EEG signals, Transformer architectures are gradually assuming a dominant role. The Adaptive Prototype-Based Subtle Transient Pattern Aware Transformer (APSTPT) framework proposed by Priyanga et al. introduces Prototype Learning and a cross-channel covariance attention mechanism, demonstrating State-of-the-Art (SOTA) performance in capturing subtle and transient pathological waveforms [6]. Furthermore, to mitigate the persistent challenge of scarce medically annotated data, frontier research has pivoted towards Generative AI and Self-supervised Learning (SSL). For example, Generative Adversarial Networks (GANs) have been employed to generate high-fidelity synthetic EEG data to augment training sets, successfully synthesizing artificial EEG data from limited recordings [7]; meanwhile, the self-supervised framework proposed by Amrani et al. demonstrates the immense potential of utilizing unlabeled data to pre-train universal feature representations [8]. Collectively, these studies propel the field towards higher precision and enhanced interpretability.

Despite the noteworthy accomplishments of the aforementioned models in experiments on specific datasets, they frequently experience substantial performance deterioration in “Cross-Subject” scenarios intended for actual clinical implementation. Recent empirical studies have quantified this disparity: research published by Amrani et al. [8] reveals that while their model achieves an accuracy of 97.07% in patient-specific testing, it plummets to 74.67% in cross-subject testing. Similarly, the classic study by Temko et al. [9] indicates that even with high-performance classifiers, the cross-subject AUC typically declines by 10–20%. This “generalization gap” is not an insurmountable physical barrier but rather stems from the extreme non-stationarity and inter-subject variability of neonatal EEG signals, specifically manifesting as “Domain Shift”. Operationally, this shift is quantified by the statistical divergence in signal properties across subjects. For instance, due to varying skull thickness and fontanel closure, the scalp potential amplitude of the same neural event can vary by a factor of 2–5 times across different infants [10], while the spectral power density of background activity exhibits distinct baseline shifts. These non-stationary variations result in differing feature distributions that standard classifiers fail to generalize across. Specifically, these disparities originate from heterogeneity at two primary levels. First, physiological developmental differences: The neonatal brain is in a phase of rapid development, where significant disparities exist in myelination, cortical thickness, and skull conductivity among infants of different gestational ages. Studies indicate that the high conductivity of the neonatal skull and open fontanels cause non-linear distortions in electric field distribution, resulting in distinctly different scalp projections of the same pathological waveform across different infants [11]. Second, signal distribution differences: The background EEG activity varies tremendously between individuals. Models are highly prone to overfitting to “identity features” specific to the training set (e.g., individual-specific baseline drifts or artifact patterns) rather than learning universal “pathological features.” Therefore, the core scientific problem that urgently needs to be addressed is the construction of a deep learning architecture capable of decoupling ‘pathological features’ from ‘individual identity features,’ thereby achieving robust detection for unseen patients.

To surmount this challenge, the present study proposes a cross-subject seizure detection architecture based on the Domain-Adversarial Spatiotemporal Network. The central hypothesis posits that an ideal diagnostic model should emulate the “de-individualized” capability of an experienced clinician, actively disregarding subject-specific identity information while accurately identifying pathological waveforms. The architecture relies on a feature decoupling mechanism that first utilizes short-time Fourier transform spectrograms, followed by independent encoding via a channel-independent convolutional neural network to capture local time-frequency textures. For spatial modeling, a bidirectional long short-term memory network performs sequential scanning along the electrode arrangement. This mechanism mathematically simulates the clinical logic of comparing hemispheric symmetry, effectively extracting deep topological dependencies. Feature integration is subsequently governed by an attention pooling layer, which adaptively weights channels to filter out background noise such as motion artifacts and precisely focus on key pathological features, thereby optimizing the signal-to-noise ratio (SNR). The core innovation of this study is the integration of a dual-head structure for adversarial learning. Through a gradient reversal layer, the model is compelled to strip away features strongly correlated with individual identity, retaining only universal pathological patterns. This approach fundamentally enhances system robustness against inter-subject variability.

## 2. Materials and Methods

### 2.1. Dataset

The dataset utilized in this algorithm comprises public medical data obtained from the Helsinki University Hospital [12]. This dataset comprises multi-channel electroencephalograms from 79 term neonates admitted to the NICU at Helsinki University Hospital. The study involving this dataset has received the necessary ethical approvals. The EEG signals were acquired using the NicoletOne vEEG System (Natus Medical, Middleton, WI, USA) at a sampling frequency of 256 Hz, with a recording duration of approximately 60 min per subject. Specifically, the duration of each window was set to 2 s. Although the raw EEG signals were sampled at 256 Hz, we downsampled them to 128 Hz during preprocessing to reduce computational complexity and filter out high-frequency artifacts. Consequently, each 2-s window corresponds to 256 sampling points in the time domain.

**Figure 1:**
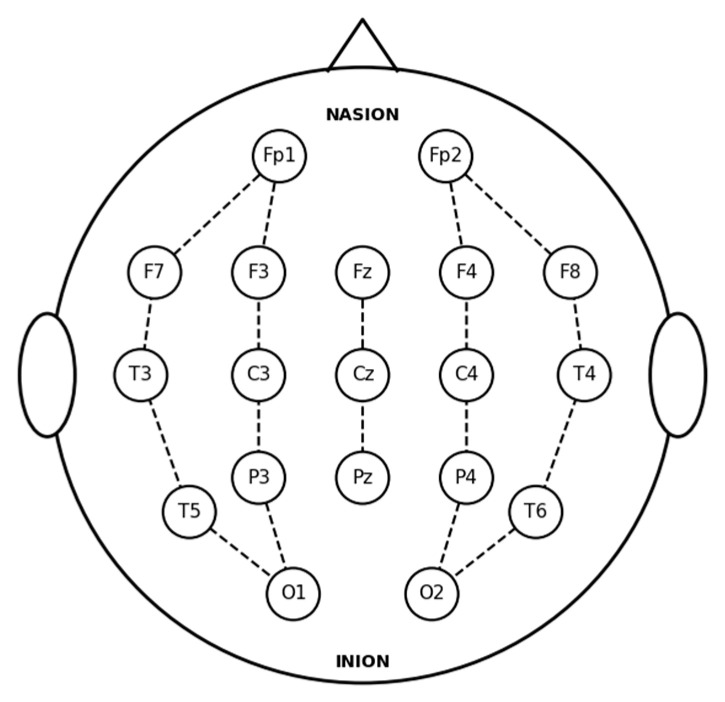
Standard 10–20 electrode placement for EEG recording. The dashed lines indicate the spatial arrangement and connections between electrode sites according to the standard 10–20 system.

**Figure 2:**
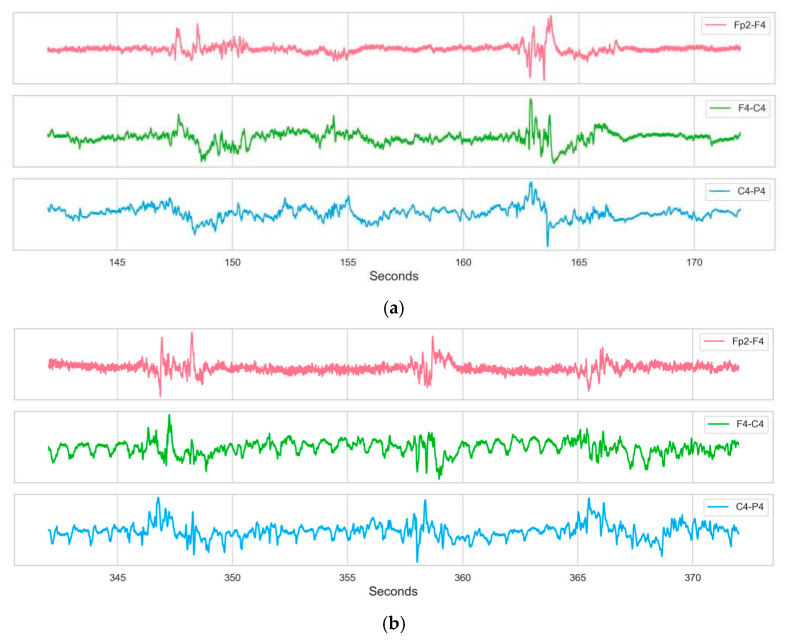
EEG activity of Sample 9: sections of non-seizure (**a**) and seizure (**b**) states.

Each recording file contains potential data from 19 electrodes, all positioned and labeled in accordance with the international 10–20 system. Figure 1 illustrates the standard 10–20 electrode layout utilized for the EEG recordings. The 18 bipolar channels derived from these electrodes are defined as follows: Fp2-F4, F4-C4, C4-P4, P4-O2, Fp1-F3, F3-C3, C3-P3, P3-O1, Fp2-F8, F8-T4, T4-T6, T6-O2, Fp1-F7, F7-T3, T3-T5, T5-O1, Fz-Cz, and Cz-Pz. Figure 2 displays the EEG activity from Subject 9, highlighting specific segments corresponding to non-seizure and seizure periods.

### 2.2. Data Preprocessing

The dataset was annotated on a second-by-second basis (1 s temporal resolution) by three independent clinical experts. A label value of 0 indicates that the expert did not observe a seizure during that second, whereas a label value of 1 indicates that the expert observed a seizure. Given the discrepancies in seizure identification among experts, this study established a Majority Voting Protocol applied to the raw annotations provided by the three independent clinical experts to construct robust supervisory signals. According to this protocol, for any given 1 s time window, a segment is confirmed as a seizure sample if at least two experts simultaneously classify it as abnormal (label 1); otherwise, the segment is uniformly rectified as a non-seizure sample (label 0). Consequently, the following definitions are provided: Let t denote the index of the sliding window (t=1,2,…,M). To ensure label consistency, the ground truth label $Y_{t}$ for the t-th window is determined by the expert consensus at the window’s central time point. The label generation rule is formalized as follows:

(1) $$Y_{t}=\left\{\begin{array}{ll} 1, & if\;\sum_{i=1}^{3}L_{i,center}\geq 2\\ 0, & otherwise \end{array}\right.$$

where $L_{i,center}$ represents the annotation of the i-th expert at the central second of the window (1 for seizure, 0 for normal).

Specifically, a sample is designated as positive only if a minimum of two experts concur on the seizure classification; otherwise, it is labeled as negative. To quantify label uncertainty and minimize the impact of label noise on model training, this study introduces the Annotation Disagreement Rate (ADR) as a metric for sample quality assessment. For a given subject, the ADR is defined as the ratio of the total duration of disagreement to the total duration of the raw recording:

(2) $$ADR=\frac{T_{disagreement}}{T_{total}}\times 100\%$$

where $T_{disagreement}$ represents the cumulative duration (in seconds) of all epochs where the expert consensus was not unanimous (i.e., $\sum_{i=1}^{3}L_{i,center}\in 1,2$). Based on this metric, a rigorous data selection strategy was implemented. To construct a class-balanced and high-quality experimental subset, 15 subjects exhibiting the highest annotation consistency were selected from the “Seizure Group” and the “Non-Seizure Group”, respectively, by ranking them in ascending order of ADR. This resulted in a total cohort of 30 subjects. This strategy effectively eliminated marginal samples with ambiguous labels, ensuring that the model was trained on data characterized by high clinical consensus. This selection strategy prioritizes label certainty. By filtering out subjects with high inter-rater disagreement (high ADR), we aim to minimize the impact of label noise on model training, ensuring the model learns from high-confidence consensus labels rather than ambiguous annotations. It is important to note that this selection criterion filters for label certainty rather than biological homogeneity. The selected subjects retain significant physiological heterogeneity in terms of gestational age and seizure morphology, ensuring that the model learns robust pathological features applicable to diverse populations rather than overfitting to ambiguous annotations.

The original dataset inherently categorizes subjects based on clinical annotations. Specifically, the 39 infants identified as having experienced at least one seizure event constituted the candidate ‘Seizure Group’, whereas the 22 infants unanimously classified as seizure-free formed the ‘Non-Seizure Group’. Table 1 presents the annotation data for representative infant EEG samples.

Given that data exhibiting significant inter-expert disagreement may compromise model performance, we selected subjects 9, 11, 13, 21, 31, 34, 36, 44, 47, 50, 52, 62, 66, 68, and 75 from the group identified as having seizures, as these subjects demonstrated minimal discrepancies in expert annotation. Concurrently, subjects 3, 10, 18, 27, 28, 29, 30, 32, 35, 37, 45, 48, 49, 53, and 55 were selected from the seizure-free group to jointly constitute the final dataset. This strategy not only enriches the diversity of the dataset but also enhances the generalization performance of the model.

**Figure 3:**
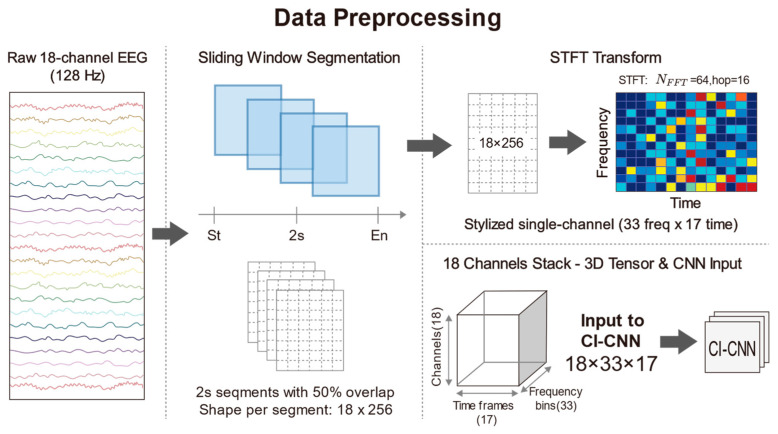
The diagram of data preprocessing.

To construct the standardized input tensors required by deep CNNs and to capture the transient seizure features inherent in neonatal EEG signals, continuous multi-channel EEG recordings were segmented into fixed-length time windows. Given that neonatal seizures typically manifest as short-duration, paroxysmal rhythmic discharges and that the signals exhibit high non-stationarity, a sliding window technique was employed to segment the preprocessed 18-channel EEG signals. Specifically, the duration of each window was set to 2 s, which corresponds to 256 sampling points in the time domain at a sampling rate of 128 Hz. To mitigate the risk of overfitting on limited medical data, we applied a 50% overlap to the sliding windows specifically as a data augmentation strategy during the training phase. Crucially, the subsequent 5-fold cross-validation is strictly subject-independent, ensuring that no correlated overlapping segments exist between the training and validation sets, thereby preventing data leakage. This short-window design also ensures that the statistical properties of the signal remain relatively stable within a localized time scale, thus satisfying the fundamental assumptions of subsequent spectral analysis. Following the subject selection and sliding window segmentation processes, the final experimental dataset comprised a total of 16,514 samples. This substantial data volume provides a robust foundation for deep model training. Furthermore, to guarantee label consistency, the class label $Y_{t}$ for each window was determined exclusively by the expert consensus at the central time point of the window. This rule explicitly resolves the classification of windows straddling seizure onset or offset boundaries: the label is strictly dictated by the state of the central second, regardless of the boundary position. This ensures that the label corresponds to the core content of the segment. We acknowledge that this central-point labeling strategy effectively acts as a duration filter. While it may exclude extremely short events (<2 s) that do not overlap with the window center, this trade-off is intentional. It prioritizes label specificity, ensuring that the model is trained on high-confidence pathological patterns rather than ambiguous transient artifacts. In the feature extraction phase, traditional time-domain analysis often fails to fully reveal the complex evolutionary patterns of seizure signals in the frequency domain. Conversely, while Power Spectral Density (PSD) estimation effectively reduces noise, it sacrifices critical temporal information through time-averaging operations, rendering it incapable of reflecting dynamic frequency changes during seizure events. In light of this, this study employed the STFT to convert one-dimensional time series into two-dimensional Time-Frequency Spectrograms, thereby simultaneously preserving both spectral characteristics and temporal evolution information. STFT was applied to each 2-s EEG segment using a Hann window to minimize spectral leakage. The number of Fast Fourier Transform (FFT) points ($N_{fft}$) was set to 64, and the sliding hop length was set to 16. No manual zero-padding was applied; boundaries were handled via standard centering. Given the sampling rate of 128 Hz, this configuration yields a frequency resolution of 2 Hz. This choice explicitly favors temporal resolution over fine-grained spectral detail, enabling the effective capture of transient high-frequency spikes while maintaining sufficient resolution for low-frequency (0.5–30 Hz) pathological slow waves. Following the STFT transformation, the complex spectrum was obtained, and the natural logarithm of its magnitude was computed to compress the immense dynamic range of EEG signals and ameliorate data distribution skewness. Consequently, each EEG window was transformed into a 3D tensor with dimensions of 18 × 33 × 17, where 18 represents the number of spatial electrode channels, 33 denotes the frequency components, and 17 indicates the time steps. This time-frequency representation not only filters out a portion of background noise but also accentuates the regions of energy concentration associated with seizure onset. To accelerate neural network convergence and eliminate amplitude discrepancies between patients, Z-score normalization was further applied to the generated spectrograms, standardizing them to a mean of 0 and a standard deviation of 1. Through the aforementioned processing pipeline, raw physiological signals were converted into feature maps encapsulating three-dimensional information—space, frequency, and time—providing high-quality input data for the subsequent deep feature extraction based on ResNet. The detailed data preprocessing procedure is illustrated in Figure 3.

### 2.3. Domain-Adversarial Spatiotemporal Network’s Entire Structure

#### 2.3.1. Model Overview

**Figure 4:**
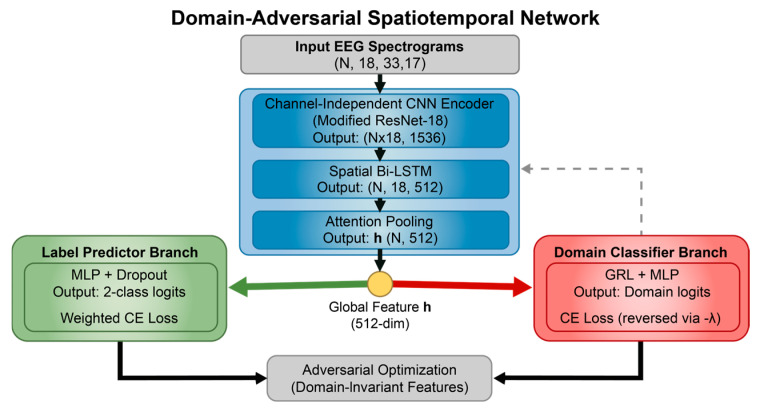
The structure of DA-STNet.

The DA-STNet constructed in this study is a deep neural network explicitly designed for cross-subject neonatal seizure detection. The network adopts a “shared backbone-dual branch” topology, aiming to concurrently process the recognition of seizure pathology and the obfuscation of subject identity. The model first utilizes a cascaded spatiotemporal feature extractor to map multi-channel EEG signals into a high-dimensional feature space. Subsequently, it introduces an adversarial mechanism via a GRL, establishing a competitive interplay between the main branch for label prediction and the adversarial branch for domain classification. This design effectively strips away individual identity information from the features while maintaining efficient feature extraction capabilities, thereby realizing a calibration-free, “plug-and-play” diagnostic solution. Figure 4 illustrates the overall structure of the proposed DA-STNet.

The backbone network of the proposed model serves as the core feature extractor for the entire system, undertaking the task of transforming raw time-frequency spectrograms into highly abstract semantic vectors. Addressing the unique “time-frequency-spatial” composite structure of EEG signals, this backbone does not employ a monolithic network structure; instead, it utilizes a cascaded fusion of CNN and Attention mechanisms [13].

CI-CNN Encoder: Considering the batch training mechanism of deep learning, the model input is a four-dimensional tensor incorporating the batch dimension. The model first reshapes the input four-dimensional tensor (N,18,33,17) into (N×18,1,33,17), dismantling the 18 electrode channels of each patient into 18 independent samples stacked along the batch dimension. The subsequent CNN shares weights across these independent single-channel spectrograms. The output of this stage is a tensor of shape (N,18,1536), representing the independent local feature representations for each of the 18 channels.

Spatial Bi-LSTM: Following the extraction of local features for each electrode, the model arranges the 18 electrodes according to a specific topological order, treating them as a sequence of length 18. A Bi-LSTM module is employed to scan across this spatial sequence. Through this processing module, the feature dimension is reduced from 1536 to 512. This operation not only compresses information but also integrates global spatial topological knowledge.

Attention Pooling: For the feature of each channel output by the Bi-LSTM, the attention module calculates a scalar score via a fully connected layer and normalizes it into a weight using a Softmax function. The model then aggregates information from all channels through weighted summation to generate a unique global feature vector. This 512-dimensional vector represents a high-level condensation of the entire EEG sample, providing the purest input for subsequent classification decisions.

The global feature vector output by the backbone network is immediately fed into two parallel task branches. The Label Predictor branch maps abstract features to the final “Seizure/Non-Seizure” diagnostic probability via a Multi-Layer Perceptron (MLP). Conversely, the Domain Classifier branch embeds a GRL at its input. This branch aims to strip away the subject identity information implicit in the features through gradient adversarial learning during backpropagation, ensuring that the model learns pathological representations that are universal across subjects rather than the specific “EEG fingerprints” of individuals.

#### 2.3.2. Channel-Independent Convolutional Neural Network (CI-CNN)

The CI-CNN Encoder module plays the critical role of a “sensory frontend” within the overall architecture. By processing each electrode channel independently via a weight-sharing convolutional network, it decouples spatial information from time-frequency textures, compelling the model to focus on learning universal pathological morphological features rather than the electrode spatial distribution patterns specific to individual patients. Its core task is to extract high-dimensional texture features from the EEG time-frequency spectrograms processed by Short-Time Fourier Transform [14].

To fully preserve the spatiotemporal-frequency characteristics of EEG signals, the model input is defined as a four-dimensional tensor $X\in R^{N\times C\times F\times T}$. Here, N represents the Batch Size during training; the spatial dimension C=18 corresponds to the standard scalp electrode leads in the international 10–20 system, designed to capture spatial dependencies across different brain regions; the spectral dimension F=33 covers the physiological frequency band of 0.5–30 Hz, aiming to encompass rhythm features with diagnostic significance; and the temporal dimension T=17 represents the number of signal evolution frames within the short time window, preserving subtle temporal dynamics. To achieve “Channel-Independent” processing, a critical tensor reshaping operation is first executed to “fold” the spatial dimension C into the batch dimension N. The transformation $T_{fold}$ is defined as follows:

(3) $$Xind=Tfold(X)\in R^{(N\cdot C)\times 1\times F\times T}$$

The geometric significance of this operation lies in the fact that the model no longer treats a sample as “one 18-channel image,” but rather as “18 independent single-channel images.” This design fundamentally severs the ability of the convolution operation to perceive spatial correlations at this stage, ensuring that feature extraction is based purely on local time-frequency textures.

The main body of the backbone network adopts the classic ResNet-18 architecture [15]. However, tailored to the low resolution and single-channel characteristics of neonatal EEG spectrograms, we performed specific lightweight modifications. First, the number of input channels in the initial convolutional kernel was adjusted to 1 to accommodate grayscale time-frequency spectrograms. Second, to prevent overfitting or gradient disappearance when processing relatively simple EEG texture features, we removed the deeper Layer 4 and the fully connected layer from the original architecture, retaining only Layer 1 through Layer 3 for hierarchical feature extraction. Building on this, this study introduces a Channel-wise Weight Sharing mechanism. Let $f_{\theta }$ denote the mapping function of the improved ResNet-18 with parameters θ. For the input of the c-th electrode channel of the n-th sample, denoted as $x_{n,c}$, the feature extraction process can be formally expressed as $z_{n,c}=f_{\theta }(x_{n,c})$. Since all C=18 channels share the same set of parameters θ, this mechanism is not only equivalent to expanding the training data volume by a factor of C, thereby enhancing the model’s generalization capability on small-sample medical data, but also endows the model with Spatial Translation Invariance. Regarding the computational scaling capability, this ‘folding’ strategy implies that the computational complexity scales linearly (O(C)) with the number of electrode channels. Unlike spatial-mixing architectures, where complexity often grows quadratically ($O(C^{2})$), our design ensures efficient scalability. Crucially, although folding increases the effective batch size by a factor of 18, the removal of the computationally intensive Layer 4 and fully connected layers effectively offsets this load, ensuring that the model’s resource consumption remains within a lightweight range suitable for clinical deployment.

Through layer-by-layer abstraction via ResNet residual blocks, the original two-dimensional spectrograms are mapped into high-dimensional feature maps. Subsequently, through adaptive average pooling and flattening operations, each channel outputs a high-dimensional vector. Specifically, the feature tensor output at the end of the backbone network contains 512 feature channels and retains 3 units of spatial information in the frequency domain. To align the features for input into the subsequent temporal module, a flattening operation is executed to unroll this tensor into a one-dimensional vector, the dimension of which is derived from the product of the channel count and the spatial dimension (512×3=1536). Therefore, for the c-th channel of the n-th sample, its feature representation can be formally defined as:

(4) $$z_{n,c}\in R^{1536}$$

The 1536-dimensional vector here serves as a highly condensed representation of the signal state for that channel within the current time window. Before transmitting the data to the subsequent module, we need to execute the inverse reshaping operation $T_{unfold}$ to restore the spatial structure of the data:

(5) $$Zout=Tunfold(z_{n,c})\in R^{N\times C\times 1536}$$

**Figure 5:**
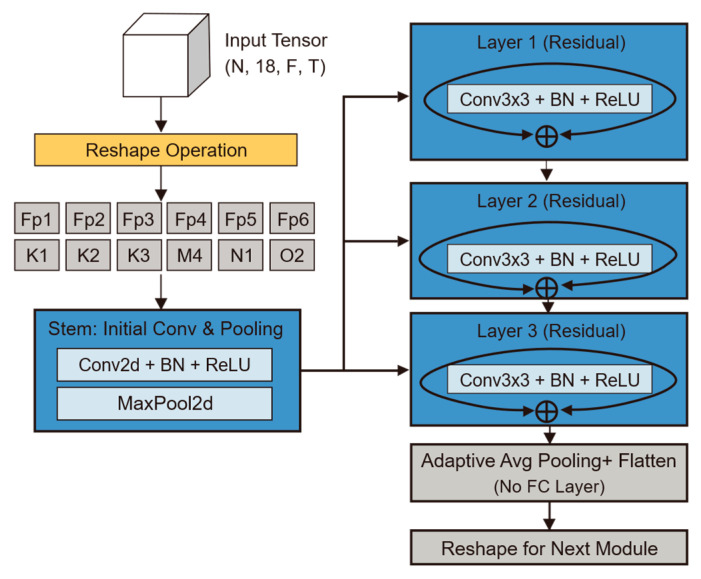
The diagram of CI-CNN.

At this stage, although the output tensor $Z_{out}$ contains features from all channels; these features remain isolated from one another. The CNN encoder is exclusively responsible for discerning the local details of each electrode, while disregarding the connections between electrodes. This establishes the foundation for the subsequent module, namely the Bi-LSTM. The detailed structure/results of this module are presented in Figure 5.

#### 2.3.3. Spatial Bidirectional Long Short-Term Memory (Bi-LSTM)

While the CNN encoder in the previous stage effectively extracted the microscopic time-frequency textures within each electrode, its “Channel-Independent” processing paradigm physically severed the inherent spatial continuity of EEG signals. Given that a seizure is essentially a global event involving abnormal synchronization and propagation across widespread brain networks, relying solely on local features makes it difficult to distinguish isolated artifacts from genuine pathological discharges [16]. To reconstruct the topological dependencies and functional coupling between electrodes, this study introduces the Spatial Bi-LSTM [17] module, aiming to fuse discrete local features into a spatial representation equipped with global context. The Spatial Bi-LSTM uses C=18 as the sequence length to scan $Z_{out}$, thereby rebuilding the brain’s topological connectivity network among the independent local features [18].

First, regarding input reshaping and sequence definition, we perform a view reshaping operation on the flattened tensor output by the CNN, denoted as $Z_{in}\in R^{(N\cdot C)\times D_{cnn}}$, to restore it to the sequence format $X_{seq}\in R^{N\times C\times D_{cnn}}.$ In this representation, the dimension C=18 is redefined as the sequence length. Based on the “Space-as-Sequence” modeling hypothesis, we treat the electrodes arranged according to the international 10–20 system as a fixed spatial sequence. This strategy is biologically inspired by the clinical ‘Double Banana Montage’ reading protocol, where neurologists scan EEG channels in specific longitudinal chains to identify propagation. Unlike Graph Neural Networks (GNNs) that require calculating dense adjacency matrices ($O(C^{2})$), this sequence-based approach maintains linear complexity (O(C)) while effectively capturing inter-hemispheric symmetry. It is important to note that while RNNs are sensitive to sequence order, ‘Permutation Invariance’ is not a requirement for standard EEG montages. Since the physical locations of electrodes are fixed under the 10–20 system, fixing the input sequence ensures consistent spatial feature extraction, analogous to how CNNs process fixed pixel grids in images. Secondly, based on the bidirectional spatial scanning mechanism [19], as well as the symmetry of the brain’s hemispheric anatomical structure and volume conduction effects, the dependencies between electrodes are inherently undirected. Therefore, we adopt a bidirectional structure to conduct a panoramic scan along the spatial axis. For the input sequence of the n-th sample, $X_{seq}^{\left(n\right)}=\{x_{1},...,x_{C}\}$, the model simultaneously maintains hidden state updates in two directions:

(6) $${\vec{h}}_{i}={LSTM}_{fwd}(x_{i},{\vec{h}}_{i-1}),\;{\overset{\leftarrow }{h}}_{i}={LSTM}_{bwd}(x_{i},{\overset{\leftarrow }{h}}_{i+1})$$

**Figure 6:**
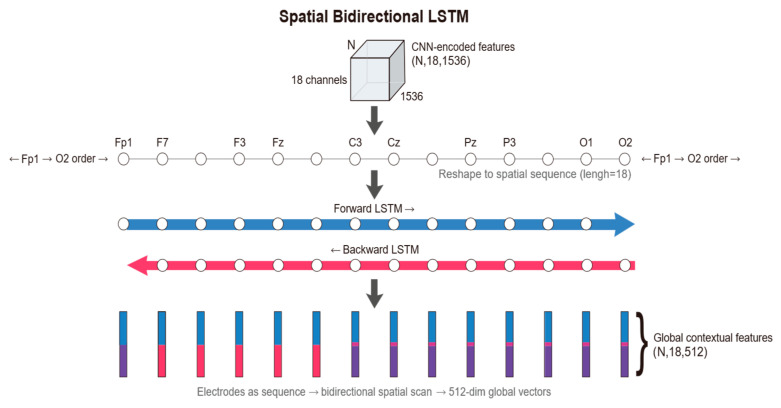
The schematic of the Spatial Bi-LSTM.

Here, ${\vec{h}}_{i}$ captures the forward spatial dependency from the frontal lobe to the occipital lobe, while ${\overset{\leftarrow }{h}}_{i}$ captures the backward contextual information. The gating mechanism internal to the LSTM unit plays a key role in this process, capable of adaptively suppressing spatial background noise during cross-hemisphere transmission. Finally, for feature fusion and output, the module generates the output vector $y_{i}=[{\vec{h}}_{i};{\overset{\leftarrow }{h}}_{i}]$ for the i-th electrode by concatenating the hidden states from both directions. In this study, we set the hidden layer dimension to 256; thus, the output feature dimension is 512. The output tensor generated through this process achieves a qualitative leap: each channel ve $H_{out}\in R^{N\times 18\times 512}$ ctor $y_{i}$ is no longer confined to a local receptive field but instead integrates global information regarding the whole-brain topological structure. These compact contextual features not only resolve the limitations of the local perspective but also provide a solid foundation for robust channel weighting in the subsequent attention pooling layer. Figure 6 illustrates the schematic of the Spatial Bi-LSTM.

#### 2.3.4. Attention Pooling Layer

Although the Spatial Bi-LSTM effectively captures the topological dependencies between electrodes, the contribution of different channels to seizure detection often varies significantly. Given that neonatal seizures frequently manifest as focal onsets, and EEG signals are susceptible to local interference from electrode detachment or motion artifacts, directly applying average pooling to all channels may dilute critical pathological information. To achieve dynamic signal-to-noise separation and global semantic compression, this study introduces an Attention Pooling Layer [20] based on the philosophy of Multiple Instance Learning (MIL). This module empowers the model to autonomously evaluate channel importance, aiming to fuse the variable-length spatial sequence features into a compact global representation through weighted integration. Attention scoring [21] and weight assignment are set as follows: The feature tensor output by the Spatial Bi-LSTM is $H\in R^{N\times C\times D}$. For the feature vector $h_{n,i}$ of the i-th electrode in the n-th sample, we first calculate its attention score via a gating mechanism-based non-linear function:

(7) $$e_{n,i}=v^{T}\tanh(Wh_{n,i}+b)$$

In this formula, W and b are learnable projection parameters used to map features into the hidden attention space; $v^{T}$ serves as the context vector, acting as a “pathological pattern expert” to evaluate the degree of matching between local features and seizure patterns. Subsequently, the Softmax function is utilized to normalize the scores, generating attention weights in the form of a probability distribution:

(8) $$\alpha_{n,i}=\frac{\exp(e_{n,i})}{\sum_{j=1}^{C}\exp(e_{n,j})}$$

The weight $\alpha_{n,i}$ directly quantifies the relative contribution of the i-th channel to the final diagnosis. This mechanism realizes a “soft channel selection” strategy: the model is capable of adaptively assigning higher weights to channels containing pathological characteristics, such as spike-and-wave discharges [22], while simultaneously suppressing those containing background noise or artifacts. This capability significantly enhances the system’s robustness within complex clinical environments. Finally, regarding global feature generation, we produce a unique global feature vector by performing a weighted summation over all channel features:

(9) $$z_{n}=\sum_{i=1}^{C}\alpha_{n,i}h_{n,i}$$

**Figure 7:**
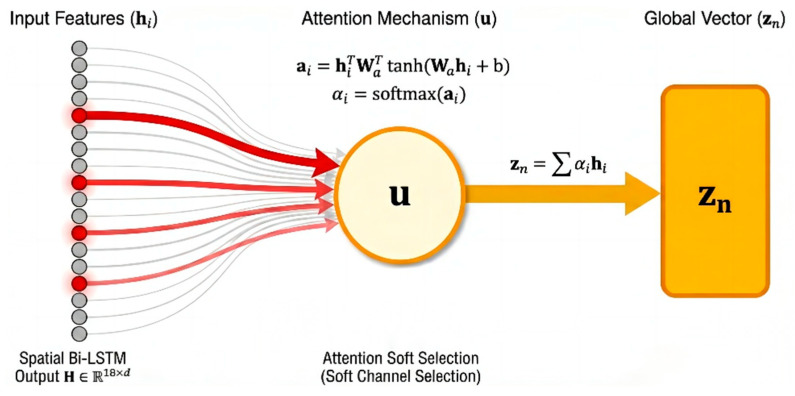
The principle of the attention aggregation mechanism. The opacity of the red arrows represents the magnitude of the attention weights.

This operation compresses the sequence data containing spatial dimensions into a fixed 512-dimensional global semantic vector. $z_{n}$ no longer contains explicit electrode location information but instead integrates the most salient pathological features of the whole brain. As the final output of the backbone network, it serves as the shared input for the subsequent Label Predictor branch and Domain Classifier branch, constituting the core vehicle for feature purification and adversarial learning within the DA-STNet architecture [23]. Figure 7 illustrates the principle of the attention aggregation mechanism.

#### 2.3.5. Label Prediction and Domain Classification Branch

At this stage, the network bifurcates into two parallel task branches: the Label Predictor ($G_{y}$) and the Domain Classifier ($G_{d}$). These two components collaboratively guide the parameter updates of the feature extractor $G_{f}(\theta_{f})$ via an adversarial mechanism. The Label Predictor branch (parameterized by $\theta_{y}$) functions analogously to a “clinician,” designed to map high-level abstract features into a diagnostic decision space. Structurally, this branch is composed of a Multi-Layer Perceptron (MLP). It employs fully connected layers coupled with ReLU activation functions to perform non-linear transformations, and incorporates a Dropout layer to mitigate the risk of overfitting. The branch ultimately yields the classification label y, distinguishing between seizure and non-seizure states. Accounting for the inherent sparsity of seizure events in neonatal EEG monitoring (i.e., class imbalance), we adopt a weighted cross-entropy loss function, denoted as $L_{y}$, as the primary optimization objective:

(10) $$L_{y}(\theta_{f},\theta_{y})=-\sum_{i=1}^{N}\left[w_{pos}\cdot y_{i}\log({\hat{y}}_{i,1})+(1-y_{i})\log({\hat{y}}_{i,0})\right]$$

**Figure 8:**
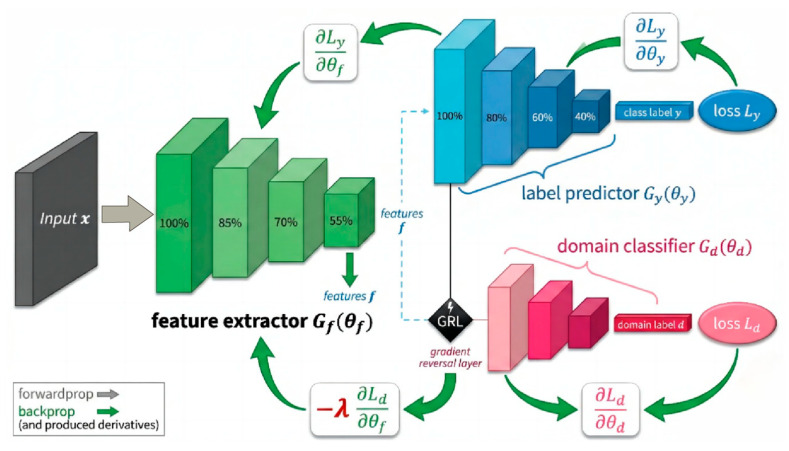
The mechanism of label prediction and domain classification branches.

Specifically, the weight parameter $w_{pos}$ is set to 2.5 to assign higher gradient importance to positive samples (seizures). This strategy compels the model to maintain high sensitivity towards subtle seizure signals during backpropagation, thereby effectively mitigating the bias introduced by class imbalance. Simultaneously, the Domain Classifier branch (parameterized by $\theta_{d}$) acts as an “identity detective,” dedicated to identifying the subject identity (domain label d) based on the extracted features f. To achieve cross-subject feature generalization, we embed a GRL at the input of this branch. During the forward propagation phase, the GRL performs an identity transformation, passing the feature f losslessly to the domain classifier to compute the domain discrimination loss $L_{d}$. Conversely, during the backpropagation phase, the GRL reverses the sign of the gradients flowing through it. As illustrated in Figure 8, the update of the backbone parameters $\theta_{f}$ is driven by the joint influence of two distinct gradient flows: the gradient from the Label Predictor, $\frac{\partial L_{y}}{\partial \theta_{f}}$, seeks to minimize diagnostic error; while the gradient from the Domain Classifier, $\frac{\partial L_{d}}{\partial \theta_{f}}$, is multiplied by a negative coefficient −λ as it passes through the GRL. This effectively converts the objective into a gradient ascent direction ($-\lambda \frac{\partial L_{d}}{\partial \theta_{f}}$), forcing the encoder to generate subject-invariant features that confuse the domain classifier. This mechanism constructs a Min-Max Game: the domain classifier attempts to accurately distinguish subjects by minimizing $L_{d}$, whereas the feature extractor, under the influence of the GRL’s reverse gradients, is forced to update parameters in a direction that maximizes the domain classification error. As training progresses, the network eventually converges to a Nash Equilibrium: the extracted features f encompass pathological information capable of minimizing diagnostic error, while simultaneously stripping away identity-specific information that distinguishes individuals. This realizes true Domain Invariance and cross-subject generalization [24]. Figure 8 illustrates the operational mechanism of the label prediction and domain classification branches.

## 3. Experiment

### 3.1. Evaluation Metrics

To comprehensively evaluate the diagnostic performance of the introduced DA-STNet in cross-subject seizure detection, we employ a multi-dimensional evaluation scheme encompassing Accuracy, Sensitivity, Specificity, F1-Score, and the AUC. Given the inherent class imbalance in neonatal EEG data—where seizure events are significantly rarer than interictal background activity—relying solely on accuracy may be misleading. Therefore, we prioritize a balanced assessment of Sensitivity and Specificity to comprehensively gauge the model’s capacity for identifying pathological patterns [25] while simultaneously minimizing false positives.

Sensitivity, also referred to as True Positive Rate (TPR), measures the model’s ability to correctly identify actual seizure events, a metric paramount for clinical safety. Specificity, or True Negative Rate (TNR), evaluates the model’s robustness in rejecting non-seizure artifacts, thereby alleviating the burden of false positives on clinicians. Accuracy represents the overall proportion of correctly classified samples across both classes. However, due to the scarcity of seizure events compared to non-seizure periods in EEG recordings, accuracy alone is insufficient for performance assessment. Consequently, we adopt the F1-Score as a critical metric to balance precision and sensitivity, ensuring the detector’s robustness against class imbalance [26]. The mathematical definitions of these metrics are as follows:

(11) $$Accuracy=\frac{TP+TN}{TP+TN+FP+FN}$$

(12) $$Sensitivity=\frac{TP}{TP+FN}$$

(13) $$Specificity=\frac{TN}{TN+FP}$$

(14) $$F1=\frac{2\cdot TP}{2\cdot TP+FP+FN}$$

Here, TP (True Positives) and TN (True Negatives) denote the number of correctly classified seizure and non-seizure samples, respectively, while FP (False Positives) and FN (False Negatives) represent the misclassified samples. To evaluate the discriminative capability of the classifier independent of specific decision thresholds, we employ the Area Under the Curve metric. A higher AUC value indicates superior generalization performance in distinguishing between seizure and non-seizure states across varying operating conditions.

### 3.2. Experimental Setup and Results

Implementation Details: The deep learning models in this study were implemented using the PyTorch 2.1.0 framework and trained in a high-performance computing environment equipped with an NVIDIA Tesla T4 GPU to accelerate computation. To optimize network weights and ensure stable convergence under non-convex loss functions, we employed the AdamW optimizer, which effectively enhances model generalization via a decoupled weight decay strategy. Regarding hyperparameter configuration, the initial learning rate was set to $1\times 10^{-4}$, the weight decay coefficient to $1\times 10^{-5}$, and the training mini-batch size was standardized to 32. To balance training sufficiency with the risk of overfitting, the maximum number of training epochs was set to 100, incorporating an Early Stopping mechanism based on validation set performance monitoring. Specifically, if the AUC metric on the validation set failed to improve for consecutive epochs, the training process was automatically terminated, and the optimal model weights were saved for subsequent evaluation. Furthermore, addressing the extreme class imbalance between seizure and non-seizure samples in neonatal EEG data, we adopted a Weighted Cross-Entropy Loss, assigning a higher penalty weight (2.5) to the minority class (seizures). This compels the model to prioritize the identification of abnormal pathological signals during the optimization process.

**Figure 9:**
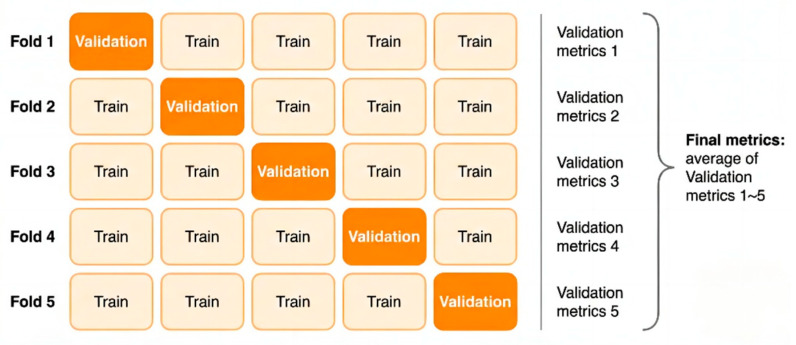
Diagram of the 5-fold cross-validation.

Regarding the dataset partitioning, we implemented a rigorous Stratified Group-wise 5-Fold Cross-Validation scheme to systematically eliminate selection bias. Crucially, to prevent data leakage, the partitioning was executed strictly at the ‘Subject ID’ level rather than the segment level. In this protocol, the cohort of 30 subjects was divided into groups, ensuring that all EEG segments belonging to the same infant were assigned exclusively to a single fold. Consequently, in each iteration, the validation set (6 subjects, 20%) consists entirely of unseen patients who are physically isolated from the training set (24 subjects, 80%). This strict Group-wise separation guarantees that no correlated signals leak across sets, confirming that the reported metrics reflect true cross-subject generalization. Figure 9 illustrates the schematic diagram of the 5-fold cross-validation process. The orange blocks represent the validation set, while the beige blocks represent the training set. In each fold, one subset serves as the validation set and the remaining four subsets serve as the training set. Final metrics are calculated as the average of the five validation metrics.

Under this strategy, the dataset is divided into five mutually exclusive subsets, each containing all data from specific subjects. This ensures that data from the same subject does not appear simultaneously in both training and validation sets, thereby effectively preventing performance inflation caused by “data leakage.” In each validation round, the model utilizes four folds (approximately 80% of the total data) as the source domain for feature learning and parameter updates, while the remaining fold (approximately 20%) serves as the target domain to evaluate the model’s adaptability to unseen individuals. This 80/20 partitioning ratio is a classical configuration in statistical learning; it ensures the model possesses sufficient data for feature extraction while reserving an adequate sample size for statistically significant performance evaluation, thus being recognized as an optimal balance between bias and variance [27,28].

Consequently, the final experimental results are not derived from a single stochastic training run. Instead, they are obtained by aggregating performance metrics from five independent cross-validation experiments and calculating their mean and standard deviation. The measured model performance and comparisons on this test set are presented in the following table.

### 3.3. Source Data Scalability and Low-Resource Bounds

**Figure 10:**
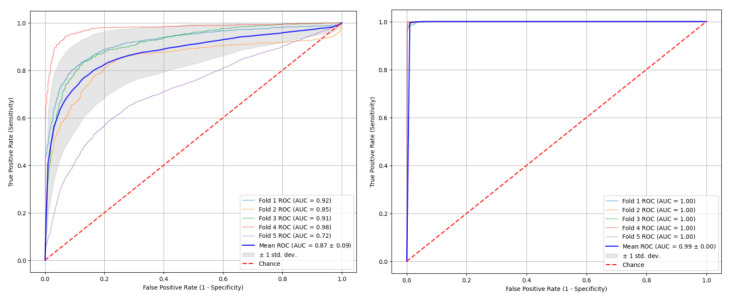
Comparison under the condition of data deficiency.

In real-world clinical environments, acquiring large-scale neonatal EEG datasets with expert frame-by-frame annotation often incurs prohibitive temporal costs and high professional thresholds. Consequently, evaluating the Data Efficiency of deep learning models under limited data conditions is crucial for assessing their feasibility for clinical deployment. Figure 10 illustrates the computational resource bounds and data efficiency results.

The impact of data scarcity is evident in the performance of the baseline model (Left Panel). The substantial divergence among the five ROC curves, coupled with the wide standard deviation region (gray shaded area, mean AUC = 0.87±0.09), indicates significant model instability. This high variance across data folds suggests that the limited dataset size hampers the baseline model’s ability to learn robust, generalizable features, rendering its performance highly dependent on the specific stochastic partitioning of the training data.

To investigate the robustness boundaries of the proposed Domain-Adversarial Network in data-scarce scenarios and to verify whether the adversarial mechanism can extract universal features more effectively under limited-sample conditions, we designed a source domain data scalability experiment. In this study, we specifically investigated the impact of key hyperparameters on model performance, aiming to enhance overall performance through systematic parameter optimization. A 5-fold cross-validation protocol was employed to benchmark performance across training and validation sets. Following each parameter adjustment, the model was retrained using this protocol, and the average of the test results across folds was calculated to comprehensively re-evaluate model performance. The core rationale of this experiment lies in maintaining a fixed Target Domain test set while progressively curtailing the training data volume of the Source Domain via a stratified random sampling strategy. This design is intended to simulate scenarios characterized by varying degrees of data scarcity. Specifically, we constructed a series of training subsets where the data volume ratio, denoted as γ, was set to 60%,70%,80%,90%, and 100% of the original source domain data, respectively. For each specific ratio γ, the model was retrained from scratch to monitor the trajectory of its generalization performance on unseen target subjects.

**Figure 11:**
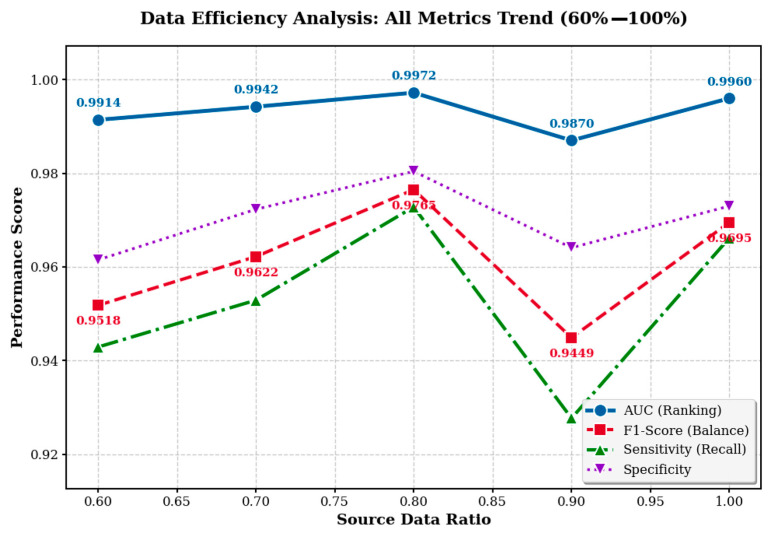
Data efficiency analysis: Metric trends across varying training set sizes.

As illustrated in Figure 11, the experimental results provide compelling evidence of the architecture’s superior Data Efficiency. Even under low-resource conditions utilizing only 60% of the source domain data, the model demonstrates robust generalization capabilities, achieving an AUC of 0.9914 and an F1-Score of 0.9518. This baseline performance indicates that, driven by the strong constraints imposed on feature distribution by domain-adversarial training, the model can rapidly capture cross-subject universal pathological features under limited sample sizes, rather than overfitting to the specific noise of a few samples. Most notably, the model achieves peak performance at an 80% data ratio, with the AUC climbing to 0.9972 and the F1-Score reaching 0.9765. This performance is even marginally superior to that obtained using the full 100% source domain data (AUC 0.9960, F1 0.9695). This seemingly counterintuitive phenomenon reveals two critical mechanisms in deep transfer learning.

Performance Saturation: The model saturates at 80% of the data volume, where further data addition yields no significant marginal gain. Implicit Regularization: Moderate data reduction introduces an implicit regularization effect during training. With full data (100%), the model tends to overfit Subject-Specific Nuances of the source subjects or memorize outlier noise points—features that are valid within the source domain but fail in the target domain. Conversely, the 80% random subset likely filters out specific interference noise, compelling the model to focus on more intrinsic, robust, domain-invariant features, thereby achieving superior generalization to the unseen target domain. In summary, the proposed architecture achieves clinical-grade diagnostic precision without reliance on massive, fully annotated datasets. This robustness against source-domain noise and high data efficiency significantly lowers the barrier to large-scale, high-quality data annotation for practical clinical deployment.

### 3.4. Ablation Study on Generalization

A comprehensive ablation study was conducted on the target domain to rigorously quantify the independent contributions of the core components within the proposed architecture—specifically, Attention Pooling and Domain-Adversarial Training—to cross-subject generalization performance. The primary objective of this experiment is to decouple two critical factors: the “enhancement of feature representation quality” and the “calibration of domain distribution shift”.

Specifically, we decompose the complete architecture into four progressive variants for comparative analysis. First, we establish a Baseline model, which comprises solely the CI-CNN and Spatial Bi-LSTM backbone. In this variant, the attention module is replaced by Global Mean Pooling for feature aggregation, and the gradient reversal coefficient is set to λ=0 to deactivate the domain adversarial branch, thereby representing the lower bound of standard deep learning performance without specialized designs. Subsequently, to validate the efficacy of the spatial weighting mechanism, we evaluate the Att variant. This model incorporates the Attention Pooling layer atop the Baseline while keeping domain-adversarial training disabled, aiming to examine the model’s capability to suppress noisy channels within a purely supervised learning framework. Third, to isolate the contribution of the domain adaptation mechanism, we construct the DA variant, which retains the GRL and domain classifier but utilizes mean pooling as a substitute for the attention layer. This setup investigates whether mere distribution alignment can sustain generalization performance in the absence of fine-grained channel selection. Finally, all the aforementioned modules are integrated to form the complete model proposed in this study (Full).

**Figure 12:**
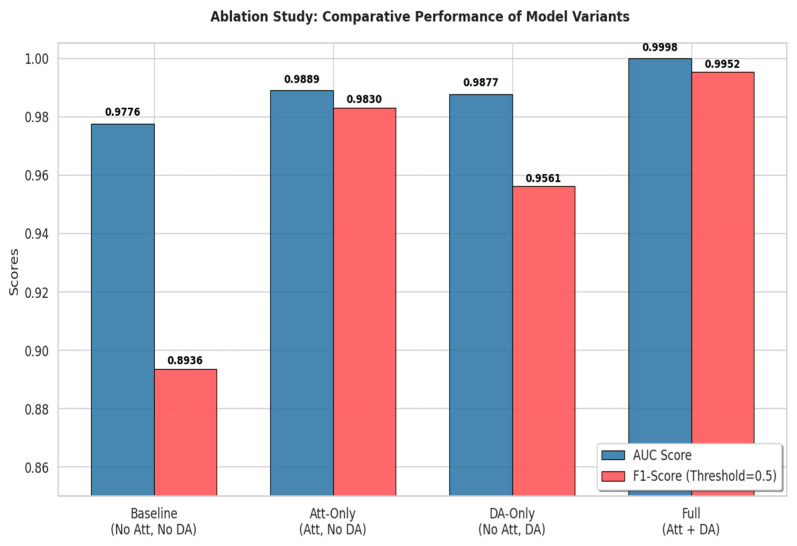
Quantitative results of the ablation study.

As illustrated in Figure 12, the experimental results demonstrate that even the Baseline model—devoid of both attention mechanisms and domain-adversarial training—achieved impressive performance, securing an AUC of 0.9776 and an F1-Score of 0.8936. This solid performance lower bound provides compelling evidence for the efficacy of the proposed “Spatial Bi-LSTM” backbone. It suggests that by treating the EEG electrode arrangement as a spatial sequence and performing bidirectional scanning, the model successfully captures inter-hemispheric topological dependencies and long-range contextual information. This explicit modeling of spatial structure constitutes a robust foundation for cross-subject transfer learning. However, the relatively lower F1-Score (0.8936) of the Baseline implies limitations regarding false positives or missed detections when analyzing subject-specific nuances or subtle signals within the target domain.

Building upon this, the variant incorporating the attention mechanism (Att-Only) solely exhibited a significant performance leap, with the F1-Score surging from the baseline’s 0.8936 to 0.9830, and the AUC improving to 0.9889. This result underscores the pivotal role of the Attention Pooling layer in signal-to-noise separation: via a soft channel selection mechanism, the model adaptively suppresses noisy channels containing artifacts while focusing on pathological regions, thereby extracting higher-quality and purer spatiotemporal features within the source domain. Simultaneously, the variant utilizing only domain-adversarial training (DA-Only) also surpassed the Baseline in terms of F1-Score, reaching 0.9561, with the AUC rising to 0.9877. This confirms that coercing the alignment of feature distributions between the source and target domains via the GRL effectively mitigates the domain shift problem. Notably, however, the F1-Score of DA-Only (0.9561) is slightly lower than that of Att-Only (0.9830), a phenomenon characteristic of domain-adversarial training. By enforcing domain invariance, the adversarial mechanism functions as a regularizer, explicitly discarding domain-specific features—which, while discriminative within the source domain, may prove detrimental to generalization on unseen samples. Consequently, this slight performance constraint is accepted as a reasonable trade-off for acquiring robust, subject-independent representations.

**Figure 13:**
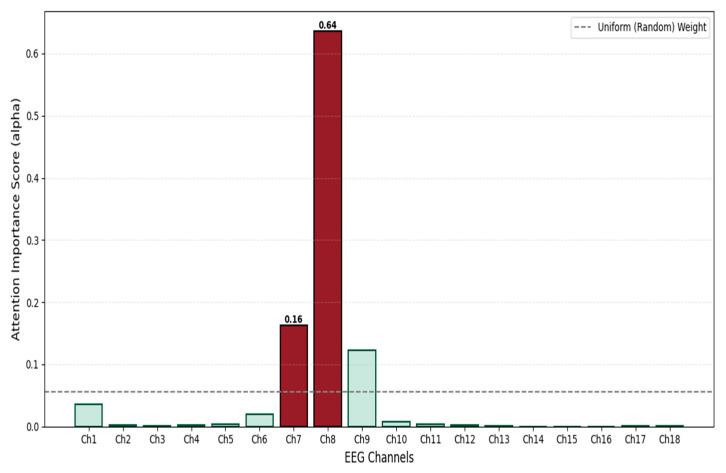
Spatial Attention Weight Distribution. The deep red and light green bars represent high and low attention weights, respectively.

To explicitly validate the claim that the model focuses on pathological regions rather than relying on speculation, we further visualized the internal distribution of attention weights ($\alpha_{n,i}$) across the 18 channels. As shown in Figure 13, the weight distribution for a representative seizure sample is highly sparse and functionally distinct. Specifically, channels Ch7 and Ch8 exhibit dominant importance scores (0.16 and 0.64, respectively), significantly exceeding the uniform baseline (dashed line). In contrast, the weights for the remaining channels containing only background activity are suppressed towards zero. This observable behavior confirms that the performance improvement is physically attributable to the model’s precise spatial focusing capability.

Ultimately, the Full model, which integrates all proposed components, achieved optimal performance, delivering a remarkable AUC of 0.9998 and an F1-Score of 0.9952. This near-perfect performance is not merely a linear superposition of individual modules; rather, it validates the potent synergistic effect between the attention mechanism and the domain-adversarial strategy. Specifically, the attention mechanism acts as a frontend filter, providing high signal-to-noise ratio (SNR) features to the adversarial training process. This empowers the domain classifier to concentrate on eliminating deep-level semantic discrepancies associated with subject identity, rather than being misled by shallow noise interference. Reciprocally, the domain-adversarial training ensures that these highly attended pathological features possess universal distributional properties across diverse subjects.

In conclusion, the Spatial Bi-LSTM establishes a robust spatial structural representation, the Attention Mechanism mitigates noise interference, and Domain-Adversarial Training overcomes distribution shifts. These three components are indispensable, collectively culminating in the model’s exceptional generalization capability in cross-subject scenarios.

## 4. Discussion

This study addresses the persistent challenge of cross-subject generalization in neonatal EEG seizure detection—specifically, the capability to maintain high diagnostic precision on unseen patient data without the need for tedious individual calibration. By proposing a spatiotemporal feature learning architecture based on DA-STNet, we have successfully constructed an end-to-end system capable of decoupling pathological features from subject identity features within complex scalp EEG signals. Experimental results demonstrate that the model achieved superior performance under strict subject-independent cross-validation, yielding an average AUC of 0.9998 and an F1-Score of 0.9952, with both sensitivity and specificity exceeding 99.4%. These findings not only validate the efficacy of the technical trajectory combining “Channel-Independent CNN, Spatial Sequence Modeling, and Adversarial Domain Adaptation,” but also mark a pivotal step towards a “plug-and-play” application paradigm for automated seizure monitoring in clinical settings.

The primary contribution of this study lies in restructuring the feature extraction paradigm for EEG signals from a systems engineering perspective. Traditional feature engineering or purely supervised learning models often fail to surmount the domain shift caused by the inherent non-stationarity of EEG signals, leading to a precipitous degradation in performance across cross-patient scenarios. In contrast, the proposed architecture effectively captures micro-level time-frequency textures via the Channel-Independent CNN, reconstructs inter-hemispheric topological dependencies utilizing the Spatial Bi-LSTM, and achieves dynamic focusing on focal seizure regions through the Attention Mechanism. Crucially, the adversarial mechanism introduced by the GRL compels the model, at a mathematical level, to learn universal seizure representations characterized by domain invariance. This “de-personalized” feature space enables the model to disregard individual anatomical variability and the heterogeneity of background activity, responding exclusively to common pathological patterns, thereby fundamentally enhancing the system’s robustness and generalization capabilities.

Despite the promising results, this study acknowledges certain limitations that delineate directions for future research. We acknowledge that the reported AUC of 0.9998 is exceptionally high. Rather than suggesting data leakage, this performance is justified by the rigorous experimental design and data quality verified in our study. First, the strictly implemented ‘Subject-Level’ validation protocol ensures zero data leakage, confirming that the model has not memorized patient identities. Second, the data selection strategy described in Section 2.2 excluded subjects with high Annotation Disagreement Rates (ADR), ensuring the model was trained and tested on ‘high-consensus’ data where pathological patterns are definitive rather than ambiguous. Therefore, the near-perfect metric reflects the model’s capability to solve the domain shift problem under high-quality data conditions. While the proposed DA-STNet demonstrates exceptional performance on this curated dataset, we acknowledge that the cohort of 30 subjects represents a controlled environment. To confirm true universal applicability and rule out any potential dataset-specific bias, future work must prioritize validation on larger, multi-center heterogeneous datasets encompassing diverse recording devices and clinical demographics. In addition, we recognize that the cascading integration of CNN, Bi-LSTM, and Attention mechanisms introduces considerable parameter complexity. While this design is essential for capturing deep spatiotemporal dependencies to ensure high diagnostic precision, it poses challenges for deployment on resource-constrained edge devices (e.g., wearable EEG caps). Future iterations of this work will focus on model lightweighting techniques, such as network pruning and knowledge distillation, to reduce computational latency without significantly compromising the model’s robust generalization capabilities. Regarding the operating thresholds and false-alarm trade-offs, the proposed model demonstrates a high degree of robustness. As evidenced in Table 2, the model achieves a Specificity of 99.58% alongside a Sensitivity of 99.47%, implying that the False Positive Rate is maintained at a negligible level (approximately 0.42%) under the current decision settings. This confirms that the near-perfect AUC is not an artifact of sacrificing specificity. Nevertheless, for practical clinical deployment, we suggest a dynamic clinical calibration strategy rather than relying on a fixed default threshold (e.g., 0.5). The operating point should be adjusted along the ROC curve to align with specific NICU priorities—such as adopting a ‘Sensitivity-First’ approach to ensure the detection of subtle pathological events. Furthermore, the integrated Attention Mechanism aids clinicians in efficiently verifying these alarms by highlighting relevant channels, thereby forming a human-in-the-loop safeguard against false positives.

In the long term, research may extend to transformer-based pure attention architectures to capture longer-range dependencies, and applying this “de-personalized” paradigm to adult epilepsy will be a crucial step towards widespread clinical application. In summary, the Domain-Adversarial Spatiotemporal Network proposed herein offers a theoretically rigorous and practically effective universal paradigm for addressing subject variability in physiological signal analysis.

## 5. Conclusions

This study proposes DA-STNet to bridge the cross-subject generalization gap in neonatal seizure detection. By leveraging domain-adversarial learning to decouple pathological features from subject identities, the architecture achieves State-of-the-Art performance with an AUC of 0.9998 and an F1-score of 0.9952 on the curated dataset. Extensive experiments substantiate the model’s superior data efficiency, achieving performance saturation with only 80% of source data. However, we acknowledge that the current cascading architecture entails high computational complexity, and the validation is limited to a controlled cohort. Consequently, future work will prioritize model lightweighting (e.g., pruning) and rigorous validation on larger, multi-center heterogeneous datasets to confirm universal clinical applicability.

## Figures and Tables

**Table 1:** Annotation data for partial infant EEG samples.

SampleID	$T_{total}$	$T_{disagreement}$	ADR (%)
9	3550	181	5.10%
11	7488	92	1.23%
13	15,416	201	1.30%
36	5082	82	1.61%
50	9850	179	1.82%
66	11,350	332	2.93%

**Table 2:** Comparison results with other epilepsy seizure detection methods.

Model	Mean Sen (%)	Mean Spe (%)	Mean AUC	F1 Score (%)
DA-STNet	99.47	99.58	0.9998	99.52
2D-CNN [29]	-	-	0.9630	-
SSL [8]	93.10	91.00	-	-
ResV2 [30]	98.36	98.91	-	98.46
LRCN [31]	84.00	99.00	0.99	-
EEG-ECG [32]	98.31	96.39	0.9752	97.95
CNN-LSTM [33]	97.80	98.20	0.98	98.00
ST-GCN [34]	98.54	98.85	0.99	98.60
DCNN [35]	90.00	91.65	0.9805	-

The symbol ‘-’ represents undisclosed model performance metrics data for which no related information has been released at present.

## Data Availability

The dataset utilized in our study is available for download at https://zenodo.org/records/1280684 (accessed on 10 October 2025). The relevant ethical approval documents can be viewed at the following link: https://zenodo.org/records/4940267/files/Ethics_approval.pdf?download=1 (accessed on 10 October 2025).
